# Machine-learning methodologies to predict disease progression in chronic hepatitis B in Africa

**DOI:** 10.1097/HC9.0000000000000584

**Published:** 2024-11-15

**Authors:** Hailemichael Desalegn, Xianchen Yang, Yi-Syuan Yen, Nega Berhe, Brooke Kenney, Geoffrey H. Siwo, Weijing Tang, Ji Zhu, Akbar K. Waljee, Asgeir Johannessen

**Affiliations:** 1 Medical Department, St. Paul’s Hospital Millennium Medical College, Addis Ababa, Ethiopia; 2 Department of Infectious Diseases, Vestfold Hospital Trust, Tønsberg, Norway; 3 Department of Statistics, University of Michigan, Ann Arbor, Michigan, USA; 4 Department of Statistics, Stanford University, Stanford, California, USA; 5 Department of Statistics, University of California at Davis, Davis, California, USA; 6 Aklilu Lemma Institute of Pathobiology, Addis Ababa University, Addis Ababa, Ethiopia; 7 Menelik II Medical and Health Science College, Addis Ababa, Ethiopia; 8 Department of Surgery, University of Michigan, Ann Arbor, Michigan, USA; 9 Center for Global Health Equity, Michigan Medicine, University of Michigan, Ann Arbor, Michigan, USA; 10 Department of Learning Health Sciences, University of Michigan, Ann Arbor, Michigan, USA; 11 Department of Statistics and Data Science, Carnegie Melon University, Pittsburgh, Pennsylvania, USA; 12 Department of Internal Medicine, University of Michigan, Ann Arbor, Michigan, USA; 13 Department of Infectious Diseases, Institute of Clinical Medicine, University of Oslo, Oslo, Norway

**Keywords:** algorithm, liver fibrosis, machine learning, sub-Saharan Africa, viral hepatitis

## Abstract

**Background:**

Little is known about the determinants of disease progression among African patients with chronic HBV infection.

**Methods:**

We used machine-learning models with longitudinal data to establish predictive algorithms in a well-characterized cohort of Ethiopian HBV-infected patients without baseline liver fibrosis. Disease progression was defined as an increase in liver stiffness to >7.9 kPa or initiation of treatment based on meeting the eligibility criteria.

**Results:**

Twenty-four of 551 patients (4.4%) experienced disease progression after a median follow-up time of 69 months. A random forest model based on a combination of available laboratory tests (standard hematology and biochemistry) demonstrated the best predictive properties with the AUROC ranging from 0.82 to 0.88.

**Conclusion:**

We conclude that combined metrics based on simple and available laboratory tests had good predictive properties and should be explored further in larger HBV cohorts.

## INTRODUCTION

International guidelines for the treatment of chronic HBV infection depend on expensive and resource-intensive tests such as viral load quantification and transient elastography (TE) to determine treatment eligibility.^1^,^2^ These diagnostic tools are rarely available in low- and middle-income countries, where most HBV-infected individuals reside. Simplification of HBV treatment guidelines, therefore, is vital to prevent disease progression to HCC, cirrhosis, and HBV-related deaths in resource-constrained settings.

Approximately 15%–40% of patients with HBV infection will experience disease progression without antiviral therapy.^3^ Predictors of HBV disease progression include sex, age, HBV viral load, ALT, and HBeAg.^4^ Little is known, however, about the utility of longitudinal indicators to predict disease progression in chronic HBV infection.

Longitudinal-derived laboratory indicators and machine-learning (ML) models have been evaluated for patients with chronic hepatitis C to predict disease progression.^5^ ML algorithms have the capacity to incorporate multiple variables, which have been collected longitudinally to predict the development of an outcome.^6^ To the authors’ knowledge, such studies have not been evaluated in chronic hepatitis B. The aim of this study, therefore, was to use ML modeling to predict disease progression in a well-characterized cohort of HBV-infected individuals in Ethiopia.

## METHODS

### Data source and patient population

In 2015, a pilot program to treat chronic HBV infection was established at St. Paul’s Hospital Millennium Medical College in Addis Ababa, Ethiopia. In total, 1303 HBsAg-positive and HIV-negative patients aged 18 years or older were enrolled. Antiviral treatment (tenofovir disoproxil fumarate) was offered based on the European Association for the Study of the Liver (EASL) 2012 criteria with some modifications, as described.^7^ The study was approved by the National Research Ethics Review Committee in Ethiopia and the Regional Committees for Medical and Health Research Ethics in Norway. All patients gave written consent to participate in the study.

**FIGURE 1:**
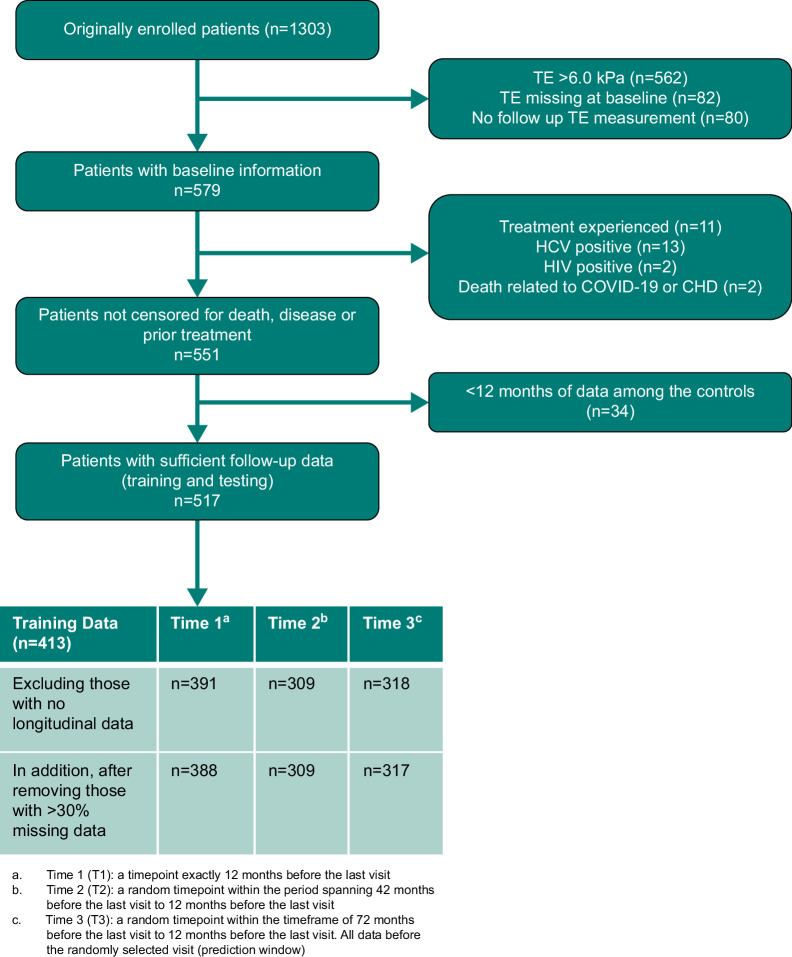
Cohort development. Abbreviation: TE, transient elastography.

For the present analytical study, we included treatment-naïve patients without liver fibrosis at the inclusion in the study, as determined by a baseline TE (FibroScan, Echosens) measurement of ≤6.0 kPa and who had at least 12 months of follow-up. Patients with HIV or HCV co-infection were excluded. Figure 1 represents how we curated our data set.

Patient evaluation at baseline included a full diagnostic work-up of laboratory tests (AST, ALT, complete blood count, bilirubin, albumin, creatinine, HBV viral load, HBsAg, and HBeAg), TE, and documentation of basic demographics. Follow-up for patients extended up to a maximum of 7 years, with laboratory tests performed at 3-month (AST, ALT, and complete blood count) or 6-month intervals (HBV viral load and HBsAg) and TE at 6-month intervals. There were, however, periods with missing data due to a patient “no-show” at the clinic or the delay in laboratory results. Patients who missed appointments were traced by the clinic staff and were categorized as lost to follow-up if they failed to respond to repeated attempts. Data were censored if the patient ceased to show up for clinic visits (loss to follow-up). In addition, data were not incorporated into the analysis after antiviral treatment was initiated.

### Outcome definition and predictor variables

#### Outcome

The primary outcome of interest was disease progression, defined as (i) an increase in liver stiffness to >7.9 kPa,^8^,^9^ or (ii) initiation of treatment based on the prespecified eligibility criteria. Liver stiffness was measured after a minimum of 2 hours of fasting, and an increase to >7.9 kPa with concomitant ALT above 200 U/L was disregarded since ALT flares may cause increased liver stiffness.^10^ Treatment was designated to patients with a liver stiffness >7.9 kPa and HBV DNA >2000 IU/mL, or ALT >80 U/L and HBV DNA >2000 IU/mL.

All patients had the same scheduled follow-up and thus the same a priori chance of reaching the outcome of interest. A proportion of patients missed appointments or were lost to follow-up, but this was independent of whether they reached the outcome of interest or not.

#### Data cleaning

Overall, we included 3 types of variables to predict disease progression: (1) baseline laboratory values, body mass index, sex, and age; (2) laboratory results from the most recent visit during the patient's follow-up; and (3) longitudinal predictors representing the distribution of each of these laboratory values as they fluctuate over time, that is, the minimum, maximum, median, mean, SD, first quantile, third quantile, coefficient of variation, IQR, skewness, kurtosis of time-variant variables, and of the slope of time-variant variables. Variables with high missingness were excluded. Refer to Appendix 1. Laboratory Parameters for additional details.

#### Sampling and model building

**FIGURE 2:**
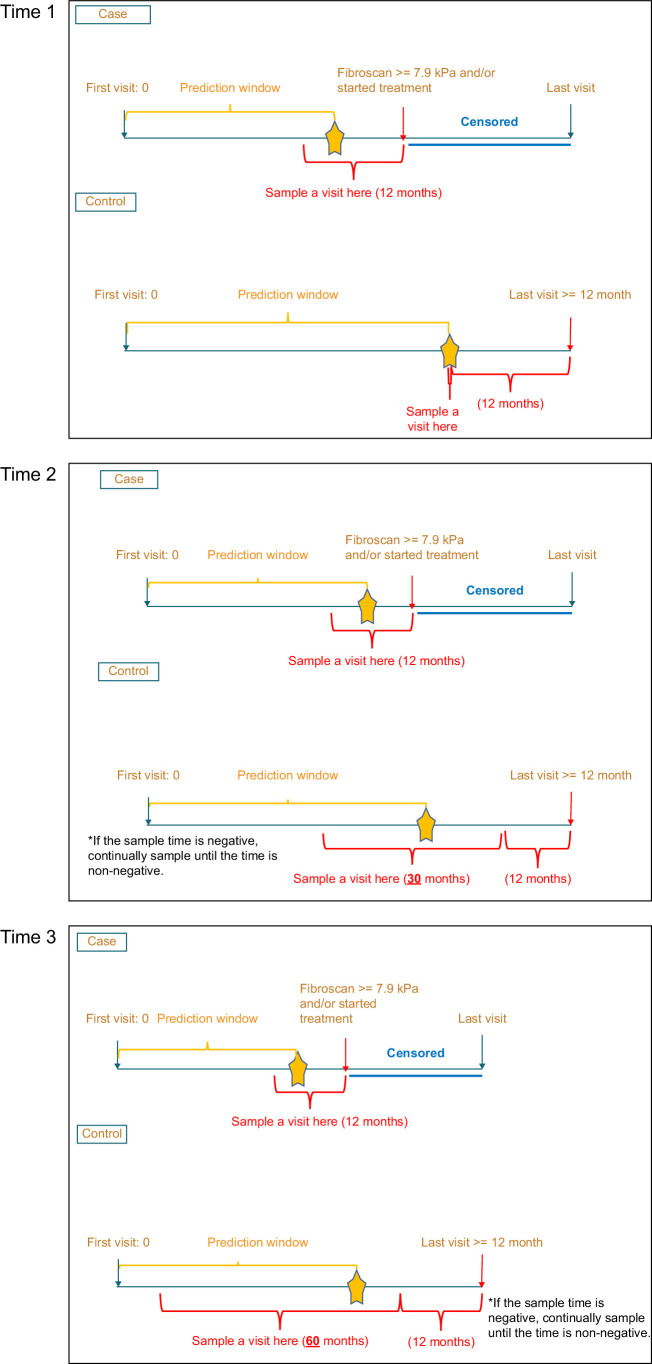
Sampling time for cases and controls for Time 1 (T1), Time 2 (T2), and Time 3 (T3).

Our objective was to predict disease progression in HBV-infected patients within a year after their clinic visit by using all available information from selected features recorded during their visit. For cases (patients with disease progression), we randomly selected a timepoint in the 12-month period before meeting the definition of treatment progression. For controls (patients without disease progression), we employed 3 distinct sampling approaches: Time 1 (T1): a timepoint exactly 12 months before the last visit; Time 2 (T2): a random timepoint within the period spanning 42 months before the last visit to 12 months before the last visit; Time 3 (T3): a random timepoint within the timeframe of 72 months before the last visit to 12 months before the last visit. All data before the randomly selected visit (prediction window) were integrated into the prediction model (Figure 2).

### Statistical analysis

**FIGURE 3:**
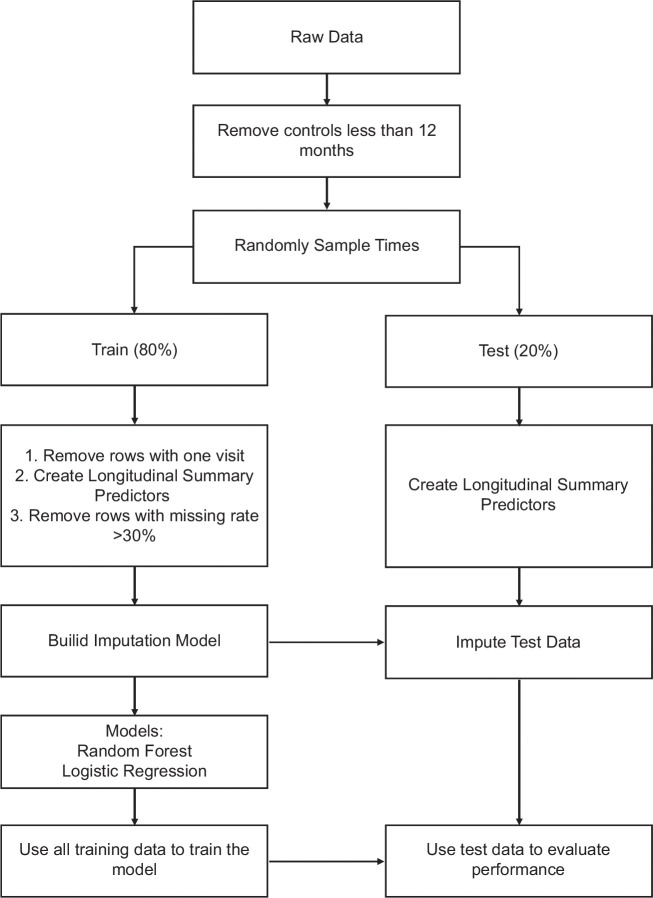
Data preprocessing and statistical methods.

Figure 3 details data preprocessing and statistical methods.

#### Data processing

The data were divided into training and testing sets using a stratified split, with 80% of the data used for training and 20% used for testing. This process was repeated 5 times, resulting in 5 pairs of training and testing sets. The stratification ensured that each pair maintained equal ratios of cases to controls.

#### Model imputation

We compared multiple imputation methods focusing on the Iterative Imputer. We primarily focused on the Iterative Imputer to impute the missing values in our data based on previous studies.^11^,^12^ It models each feature with missing values as a function of other features. We fixed the imputation parameters to reduce the computational cost using the scikit-learn 1.2.2 Package in Python (3.8.8).

#### Model building

Random forest (RF) and penalized logistic regression were employed. For RF, to reduce the computational cost, we fixed parameters (number of estimators: 5000; minimum sample split: 2; criterion: gini; and minimum sample leaf: 1). The RF method was performed in scikit-learn 1.2.2 Python (3.8.8). For penalized logistic regression, we used the LASSO penalization with 5-fold cross-validation to tune the hyperparameter. We used R software 4.1.3 with the glmnet package.

#### Model evaluation

##### AUROC

The models’ discriminatory performance was evaluated by computing the AUROC, which captures the overall performance of the model across a range of thresholds. AUROC estimates were averaged across the 5 rounds of model training and evaluation, whereby each round used a separate pair of training and test sets. The AUROC value was then calculated using the same methodology for 3 different sample times (T1, T2, and T3). The best cutoff point was determined by identifying the maximum Youden index point, which allows for the identification of an optimal cutoff point that maximizes the discriminatory power of the model.^13^

##### Brier score ​​​

Brier scores capture both calibration and discrimination and were reported as an overall measure of model performance.^14^ Brier scores range from 0 to 1, where 0 signifies perfect accuracy and better model performance compared with a higher score.^15^ Brier scores were obtained for each round of model training and evaluation.

##### RF feature importance

To assess the importance of each predictor, we conducted an evaluation within the RF model. The RF consists of multiple decision trees, where at each internal node, a specific feature is used to determine how to divide the data set into 2 subsets with similar responses.^16^,^17^ The relative importance of each predictor was determined by calculating the mean and SD of the accumulation of the gini impurity decrease across all the trees in the RF. This approach allows us to quantify and compare the contributions of different predictors in the model.

##### Partial dependence plots for RF

Partial dependence plots were generated to depict predictor influence on outcomes, involving RF model fitting, predicting, and averaging across patients for each predictor value, constructing comprehensive relationships between predictors and outcomes.

## RESULTS

### Baseline characteristics and disease progression

The average age of the study participants was 33.1 years (SD: 9.5), and 44.6% were men. The average baseline liver stiffness was 4.66 (SD: 0.84), as expected based on the selection criteria. The average body mass index was 23.4 kg/m^2^ (SD: 3.89). Only 5.1% of the participants reported a family history of HCC, while 4.2% had a history of alcohol abuse, and 6.5% reported khat abuse. Table 1 presents summary statistics for baseline laboratory measurements as a function of follow-up time: minimum of 12 months (n = 510), 36 months (n = 404), 60 months (n = 308), and 72 months (n = 170).

**TABLE 1:** Baseline characteristics of patients with a minimum of 12, 36, 60, and 72 months of follow-up time

	12 mo minimum (n = 510)	36 mo minimum (n = 404)	60 mo minimum (n = 308)	72 mo minimum (n = 170)	All (n = 551)
Age, mean (SD)	33.3 (9.6)	34.1 (9.8)	34.2 (9.5)	34.2 (9.4)	33.1 (9.5)
Men, n (%)	229 (44.9)	178 (44.1)	135 (43.8)	82 (48.2)	246 (44.6)
Family history of HCC, n (%)	25 (4.9)	19 (4.7)	10 (3.3)	7 (4.1)	28 (5.1)
Alcohol abuse, n (%)	20 (3.9)	12 (2.9)	10 (3.3)	5 (2.9)	23 (4.2)
Khat abuse, n (%)	34 (6.7)	29 (7.2)	22 (7.1)	13 (7.7)	36 (6.5)
BMI, mean (SD)	23.5 (3.87)	23.7 (3.89)	23.6 (3.95)	23.7 (3.63)	23.4 (3.89)
Baseline laboratory values, median (IQR)					
Albumin (g/dL)	45 (41–50)	45 (41–50)	45 (41–50)	45 (41–50)	45 (41–49.5)
ALT (IU/L)	22 (17–30)	22 (17–29)	21 (17–29)	21 (16–29)	22 (17–30)
AST (IU/L)	23 (19–29)	22 (19–28)	22 (19–28)	23 (19–28)	23 (19–29)
Creatinine (mg/dL)	0.8 (0.7–0.9)	0.8 (0.7–0.9)	0.8 (0.7–0.9)	0.8 (0.7–0.9)	0.8 (0.7–0.9)
TE (kPa)	4.8 (4.03–5.3)	4.8 (4–5.3)	4.8 (4–5.3)	4.8 (4.1–5.3)	4.8 (4.1–5.3)
Hemoglobin (g/dL)	15.1 (14–16.4)	15 (14.1–16.4)	15 (14.1–16.4)	14.9 (14–16.3)	15.1 (14–16.5)
Platelets (×10^9^/L)	292 (252–336)	290 (251–334)	287 (252–330)	285 (245–325)	292 (252–336)
HBV DNA (IU/mL)	917 (217–5.02e+03)	954 (228–4.92e+03 )	888 (230–4.68e+03)	938 (276–4.25e+03)	894 (217–4.6e+03)
HBV DNA (Log 10)	2.96 (2.34–3.70)	2.98 (2.36–3.69)	2.95 (2.36–3.67)	2.97 (2.44–3.63)	2.95 (2.34–3.66)

*Note:* Missing values were excluded.

Abbreviations: BMI, body mass index; TE, transient elastography.

Out of the total sample (n = 551), 4.4% (n = 24) had experienced disease progression after a median follow-up time of 69 (5.75 y) months after the baseline assessment. Supplemental Table S1, http://links.lww.com/HC9/B89, provides additional details.

### Model performance

The average AUROC and its corresponding 95% CIs for the prediction of disease progression within 12 months using RF for T1, T2, and T3, respectively, were 0.88 (0.80, 0.97), 0.84 (0.69, 0.97), and 0.82 (0.69, 0.94). For Lasso logistic regression, the results were 0.60 (0.48–0.72), 0.54 (0.45, 0.59), and 0.52 (0.48,0.56). The AUROC values were computed over 5 random splits.

For comparison, we evaluated the performance characteristics of the aspartate aminotransferase-to-platelet ratio index and Fibrosis-4, which are established markers recommended for use in the management of patients with hepatitis B. The AUROC of these 2 markers at different time points is provided in Supplemental Table 2, http://links.lww.com/HC9/B89.

The sensitivity and specificity across 3 different sample times, T1, T2, and T3, are reported in Table 2. Among the multiple splits (5 described above), we chose to use the RF model that demonstrated the closest agreement with the average performance observed across all 5 splits. The resulting misclassification table for this specific split, which excluded patients with <12 months of records from the test sample, can be found in Table 2.

**TABLE 2:** Misclassification table for one random split

Sample time (mo)	Test sample (n)^a^	Event proportion	AUROC	Brier score	Best cutoff	Specificity	Sensitivity
T1	104	4.36%	0.92 95% CI (0.87–0.97)	0.03	0.20	0.96	0.75
T2			0.84 95% CI (0.77–0.91)	0.05	0.14	0.83	0.75
T3			0.810 95% CI (0.74–0.86)	0.05	0.06	0.62	1.0

^a^ Excluding patients with <12 months follow-up.

### Feature importance

**FIGURE 4:**
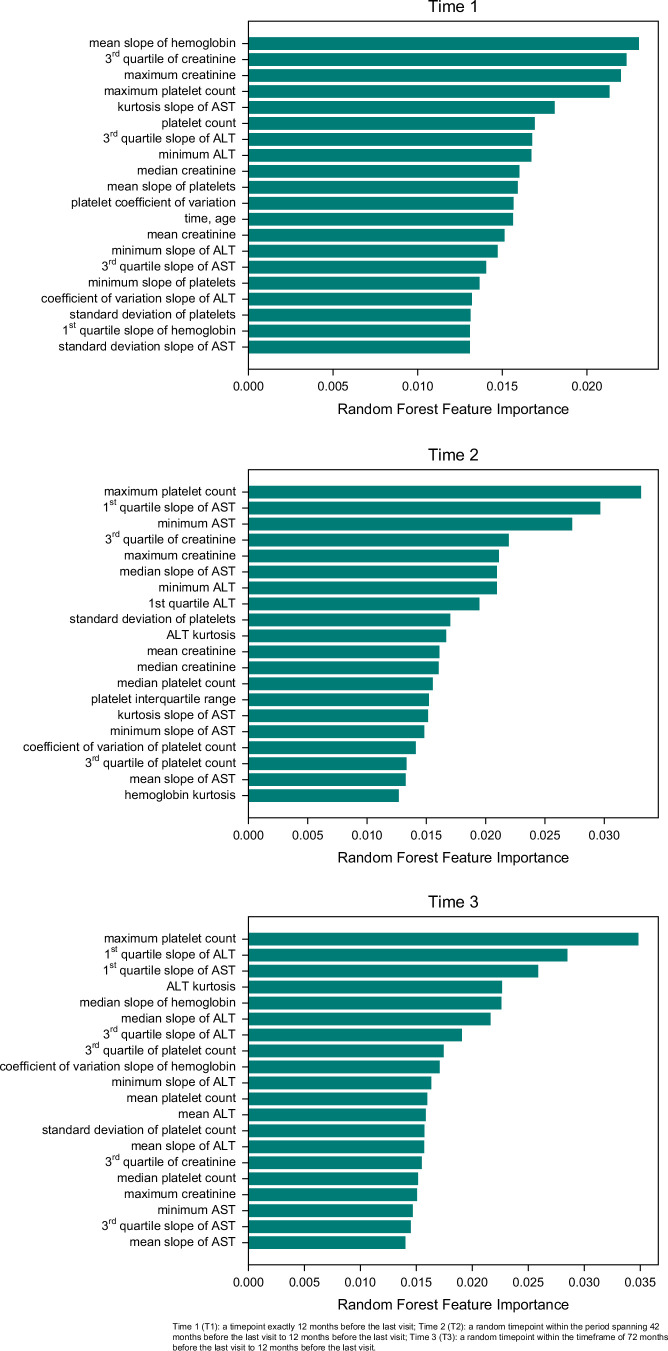
Feature importance plots for Time 1 (T1), Time 2 (T2), and Time 3 (T3).

The impact and significance of predictors in the RF model were analyzed in our study. We present the variable importance plots for 3 different sample times in Figure 4. These plots provide insights into the relative importance of variables in distinguishing disease progression.

For T1, the 5 most influential variables for predicting disease progression were the mean slope of hemoglobin, third quartile of creatinine, maximum creatinine, maximum platelet count, and kurtosis slope of aminotransferase. In the case of the T2, the 5 most influential variables were: maximum platelet count, first quartile slope of AST, minimum AST, third quartile of creatinine, and maximum creatinine. Lastly, for T3, the 5 most important variables were: maximum platelet count, first quartile slope of ALT, first quartile slope of AST, ALT kurtosis, and median slope of hemoglobin.

To better understand the influence of individual predictors on the probability of disease progression, we employed partial dependence plots in our analysis. These plots, depicted in Figure 5, showcase the effects of selected important variables in the RF model. We present the partial dependence plots for the top 5 predictors, determined by their variable importance, in each model.

FIGURE 5Partial dependence plots for the random forest model of T1, T2, and T3.

**Figure:**
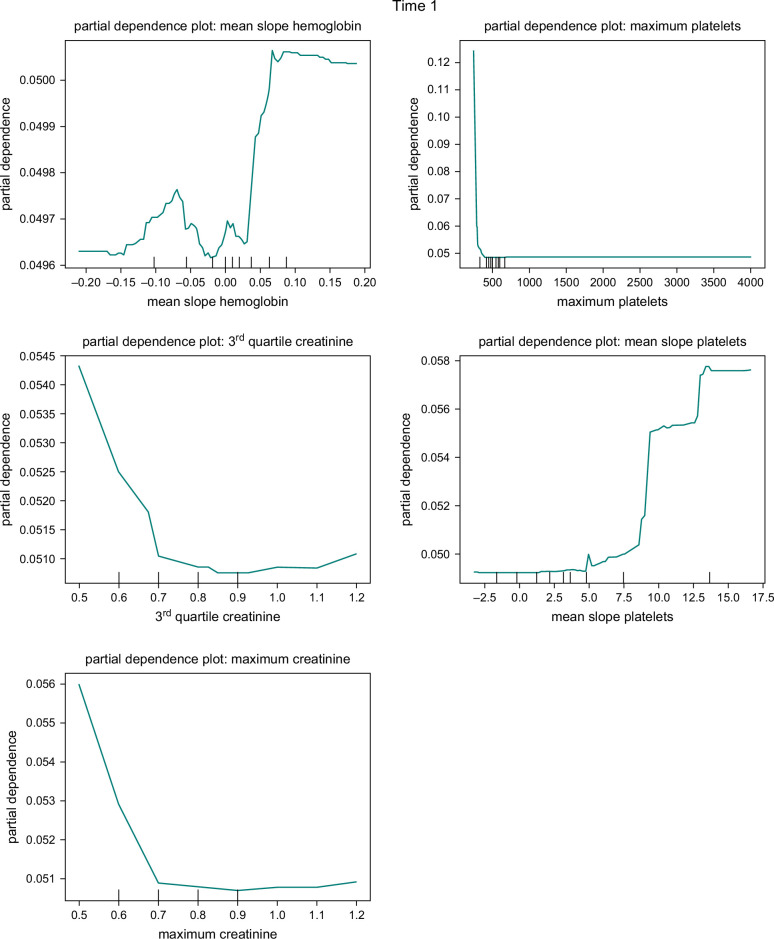

**Figure:**
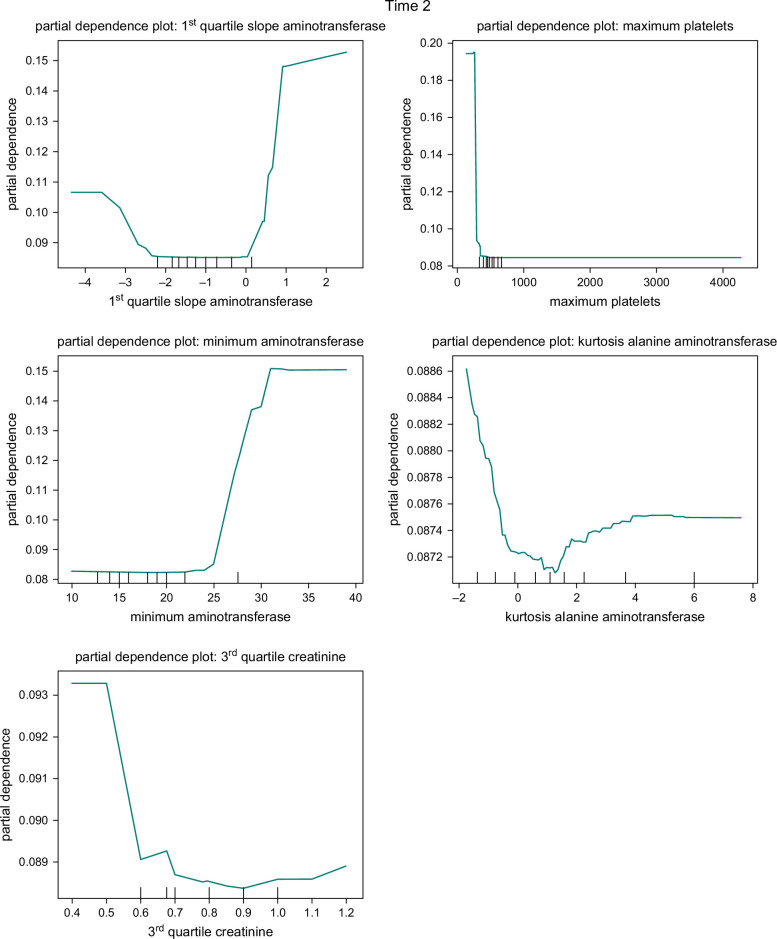

**Figure:**
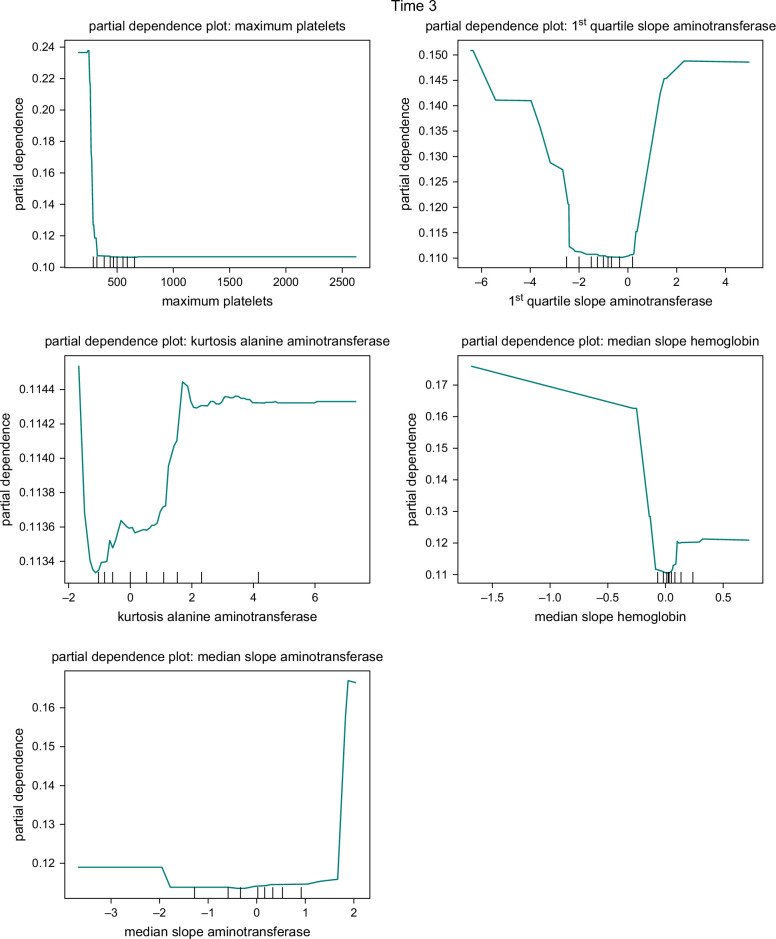

## DISCUSSION

In this study we examined if prediction models informed by longitudinal standard laboratory tests could predict the risk for disease progression in chronic hepatitis B. Interestingly, we showed that ML models could identify patterns associated with disease progression that are not previously described. Functions of simple and available laboratory markers, such as hemoglobin, platelets, AST, and ALT, appeared to be useful tools for identifying patients at elevated risk. While these findings cannot be directly translated to practical patient management, our study provides evidence that cheap laboratory markers can be useful alternatives to the traditional and more expensive tools recommended in international HBV guidelines.^2^ In low- and middle-income countries, where access to TE and viral load testing is limited, these models could be leveraged as a practical and cost-effective means of predicting disease progression. We believe our findings should be further explored in larger HBV cohorts, with the aim of identifying thresholds and algorithms for practical patient management in resource-constrained settings.

Our study team previously assessed the TREAT-B score and the World Health Organization (WHO) 2015 guideline to select patients who require early treatment. The TREAT-B score had an AUROC of 0.73 (0.68–0.78), and the WHO guideline of 0.61 (0.56–0.66), with a sensitivity of 53% for TREAT-B and 26.8% for the WHO guideline.^18^ Our current model had an AUROC of 0.88, 0.84, and 0.82 (for T1, T2, and T3, respectively), which—although not directly comparable—outperformed both TREAT-B and the WHO guidelines in this setting. Notably, our model also outperformed baseline aspartate aminotransferase-to-platelet ratio index (AUROC: 0.64) and Fibrosis-4 (AUROC: 0.51), which are markers commonly recommended in low- and middle-income countries based on their availability and low cost. See Supplemental Materials for additional performance metrics.

Many laboratory values do not exhibit a linear relationship with the development of liver fibrosis. In our study, variables like the mean slope of hemoglobin may exhibit a nonlinear trend, with a decrease followed by a significant increase. Consequently, relying solely on individual results may provide limited information, as the trends observed in longitudinal data are often more informative in clinical practice.^5^ Further studies from larger HBV cohorts are warranted to explore the utility of longitudinal laboratory testing in patients with chronic hepatitis B.

The main limitation of this study was the small sample size and low number of study participants with disease progression, which could affect the utilization of standard ML methods. Moreover, missing data impaired the data quality, and in particular, missing liver stiffness measurements since this affected the ability to ascertain the precise timing of the outcome. In addition, the data used in this study were from a single study site; hence, external validation of the models in larger prospective cohorts is required. Finally, the definition of liver fibrosis progression used in our study (increased liver stiffness from ≤6.0 to >7.9 kPa) has not been formally validated against liver biopsy. However, it is in line with previous studies using TE to define fibrosis progression,^19^ and is supported by various studies showing that increased liver stiffness is associated with liver-related morbidity and mortality.^20^,^21^

In conclusion, we used ML models to identify predictors of disease progression in chronic hepatitis B. Notably, combined metrics based on simple and available laboratory tests had good predictive properties and should be explored further in larger cohorts.

## Supplementary Material

SUPPLEMENTARY MATERIAL
