# Supplementary material for: Machine-learning methodologies to predict disease progression in chronic hepatitis B in Africa
Source: Hepatol Commun. 2024 Nov 15;8(12):e0584. doi: 10.1097/HC9.0000000000000584 (PMC11567701; doi:10.1097/HC9.0000000000000584)
Supplement: SUPPLEMENTARY MATERIAL [file hc9-8-e0584-s001.docx]

**Supplemental Materials**

Laboratory Parameters

Several variables are highly dependent of each other (height, weight and BMI) and have a high missing rate (start reason 97.8%, HIV status 99.27%), so we removed them. Overall, we included 3 types of variables to predict disease progression: (1) baseline measurements of the albumin lab value, BMI (calculated as weight in kilograms divided by height in meters squared), sex and age at the prediction time; (2) laboratory results from the most recent visit during the patient’s follow up; Hgb, platelet count, ALT, AST and creatinine; and (3) longitudinal predictors representing the distribution of each of these lab values as they fluctuate over time, i.e. the minimum, maximum, median, mean, standard deviation, first quantile, third quantile, coefficient of variation, interquartile range, skewness, kurtosis of time-variant variables and of the slope of time-variant variables, defined as the ratio of difference of the longitudinal predictor and the time gap between two subsequent visits*.*

Supplemental Table 1. ALT and HBV DNA by category at baseline

| **Category** | **Number of Patients (Total)** | **Percentage (%)** |
| --- | --- | --- |
| ALT <1x ULN | 492 | 89.29 |
| ALT 1-2x ULN | 54 | 9.80 |
| ALT 2-5x ULN | 5 | 0.91 |
| ALT >5x ULN | 0 | 0.0 |
| HBV DNA Detected - <2000 | 352 | 63.89 |
| HBV DNA 2000-20,000 | 130 | 23.59 |
| HBV DNA >20,000 | 69 | 12.52 |

ALT, alanine aminotransferase; ULN; upper limit of normal; HBV, hepatitis B virus; DNA, deoxyribo nucleic acid.

Supplemental Table 2. Diagnostic properties of APRI and FIB-4

|  | ***APRI*** | | | ***FIB-4*** | | |
| --- | --- | --- | --- | --- | --- | --- |
|  | ***Time 1*** | ***Time 2*** | ***Time 3*** | ***Time 1*** | ***Time 2*** | ***Time 3*** |
| ***AUROC*** | *0.641* | *0.643* | *0.657* | *0.514* | *0.491* | *0.520* |
| ***Best Cutoff*** | *0.155* | *0.294* | *0.188* | *0.427* | *1.280* | *0.413* |
| ***Sensitivity*** | *1.000* | *0.524* | *0.826* | *0.818* | *0.143* | *0.783* |
| ***Specificity*** | *0.287* | *0.804* | *0.459* | *0.395* | *0.957* | *0.340* |

APRI, aspartate aminotransferase to platelet ratio index; AUROC, area under the receiver operating characteristic curve.
